# Patient and Parent Experiences of Care at a Pediatric Gender Service

**DOI:** 10.1089/trgh.2018.0016

**Published:** 2018-12-28

**Authors:** Michelle A. Tollit, Debi Feldman, Gabrielle McKie, Michelle M. Telfer

**Affiliations:** ^1^ Department of Adolescent Medicine, Royal Children's Hospital Gender Service, Melbourne, Australia.; ^2^ Murdoch Children's Research Institute, Melbourne, Australia.; ^3^ Melbourne Graduate School of Education, The University of Melbourne, Melbourne, Australia.; ^4^ Western Health, Melbourne, Australia.; ^5^ Department of Paediatrics, The University of Melbourne, Melbourne, Australia.

**Keywords:** clinical care, gender dysphoria, transgender, access to care, model programs, gender identity

## Abstract

**Purpose:** To explore experiences of care at the Royal Children's Hospital Gender Service (RCHGS).

**Methods:** A total of 114 parents and 52 patients of the RCHGS completed an experience of care survey.

**Results:** Most participants highly rated elements of the family-centered care and multidisciplinary team at RCHGS. The majority were satisfied with the RCHGS (parents: 88%, patients: 92%) and would recommend the service (parents: 95%, patients: 89%). Reductions in distress after participation in RCHGS were noted. Wait time was an area of dissatisfaction. Ideas for improvement concerned information giving, family support provision, and improving access to care.

**Conclusion:** This study affirms the multidisciplinary family-centered model used at RCHGS.

## Introduction

The Royal Children's Hospital Gender Service (RCHGS) adopts a family-centred approach to its gender-affirming health care, which acknowledges and supports the gender identity^1^ of gender diverse children and adolescents up to age 17 from Victoria, Australia. The RCHGS provides assessment, support, and offers developmentally appropriate treatment pathways. For prepubertal children, assistance is provided to develop safe and supportive gender-affirming environments at home and school. Once puberty starts, options for medical treatment include puberty suppression and gender-affirming hormones.^2^ Referrals to RCHGS have dramatically increased since its single initial referral in 2003.^3^ In 2016, >220 new referrals were received, and the service has rapidly become the largest publically funded multidisciplinary pediatric gender service in Australia. The RCHGS follows international best practice guidelines in the support and treatment for gender diverse children and adolescents,^4,5^ and consists of a multidisciplinary team, including mental health specialists (psychologists and psychiatrists), pediatric medical specialists (pediatricians, adolescent medicine physicians, and pediatric endocrinologists), a clinical nurse consultant, a speech therapist, and fertility experts, as well as support from administrative staff. A multidisciplinary approach is recommended^4^ due to the complex psychological, social, and physical needs of this group.

Gender diverse individuals experience high rates of depressive and anxiety symptomatology, self-harm, and attempted suicide,^6–10^ likely to be largely related to the negative impact of societal discrimination and prejudice.^6–8^ Access to appropriate health care is a putative mechanism contributing to improved outcomes for this population. When provided with specialist multidisciplinary gender-affirming health care, the quality of life of gender diverse individuals matches that of the general population.^11^ Likewise, a study of gender diverse adults found those who had access to medical care they desired were among those with the lowest proportion of depressive symptoms.^7^ The highest proportion with clinically relevant depressive symptoms were those who desired medical treatment but had not commenced treatment,^7^ suggesting that the timely delivery of services is a determinant of mental health outcomes.

Measuring the subjective experience of health care can be a useful way to measure outcomes and drive service improvement. Patient satisfaction is increasingly seen as an integral part of quality care and is often a performance indicator within health services.^12^ Satisfaction with a health service is also associated with many benefits,^13^ including improved physiological and psychological outcomes for some patients.^14,15^ This link is demonstrated by Erasmus and colleagues^16^ who assessed patient satisfaction with an adult gender service in Melbourne, Australia, and found 88% of patients were satisfied with the service while reductions in distress having attended the clinic were noted. Despite these links, there is limited research evaluating experiences of care at gender services in pediatric settings from the perspective of adolescents and parents. We aim to evaluate the experiences of a multidisciplinary family-centered gender service among adolescent patients and parents of the RCHGS.

## Methods

### Setting and participants

The RCHGS is based at The Royal Children's Hospital (RCH): a large tertiary pediatric hospital in Melbourne, Australia. The patient sample in this study comprised current patients (as of January 2016) of the RCHGS aged 12 years or older. The parent sample was parents of current patients regardless of patients' age. Study eligibility included having sufficient English to complete surveys. Parents from 150 families were invited to participate, as were 93 patients.

### Procedure

Approval for this study was granted from the RCH Human Research Ethics Committee (HREC #35273). A list of eligible participants was generated using the RCHGS patient registry. As participants were recruited from a clinical service, parents of RCHGS patients were initially sent an introductory letter from the RCHGS Director advising them that their contact details and demographic information would be provided to the researchers to facilitate being contacted for this study. The letter asked parents to notify the director if they did not agree to this. Two patients who did not have a parent involved in their care were excluded. The contact details of parents who did not opt out were then provided to the research team who subsequently phoned parents and requested that parents nominate one parent per family to participate in the study, provide permission for their child to be surveyed if they met age criteria, and provide an e-mail address to facilitate survey administration.

Data were collected using a secure web-based application, REDCap,^17^ hosted at Murdoch Children's Research Institute. Parent and patient information letters and survey links were e-mailed to parents; parents of patients aged 12 years or older were asked to forward the patient's letter and survey to their child if they agreed to them participating. In some instances, patients aged 18 years or older provided consent for themselves. Participants completed online surveys, or pen-and-paper or telephone surveys if preferred. Survey completion was considered implied consent. Surveys took ∼10 min to complete and were undertaken during March–April 2016. After survey completion, information about support services for gender diverse patients and families was provided.

### Measures

A separate parent and a patient survey was used to measure the following.

#### Demographics and appointment history

Participant demographic information, including age, gender identity, and postcode, was collected using open-ended questions. Information about country of birth (Australia/another country), whether English was spoken as a first language (yes/no), and information about RCHGS appointment history (months on wait list and number of appointments) was also collected.

#### Experiences of care

An instrument measuring patient satisfaction with adult gender services in the United States, United Kingdom, and Australia^12,16,18,19^ was adapted with permission from the original author. In this study, experiences of care were assessed by a 32-item parent instrument and a 30-item patient instrument. Participants reported on the health care received using a five-point scale (“very poor” –“excellent”), distress levels before first appointment and at the time of survey completion on a four-point scale (“no problem”–“severe”), how helpful clinicians were on a four-point scale (“very unhelpful”–“very helpful”), satisfaction with family-centered care and administration on a five-point scale (“very dissatisfied”–“very satisfied”), and if participants would recommend the service to a friend/relative with similar concerns (five-point scale: “strongly disagree”–“strongly agree”). Open-ended questions about helpful or beneficial elements of care, areas for improvement/change, additional services, and further comments were included.

### Analysis

Quantitative data analysis was undertaken by coauthor G.M. in STATA (version SE 13.0). For analyses, responses to satisfaction items were collapsed to three categories: very dissatisfied/dissatisfied, neither dissatisfied nor satisfied, satisfied/very satisfied. Helpfulness responses were collapsed into unhelpful/very unhelpful versus helpful/very helpful.

Frequencies and percentages of categorical data and means and standard deviations of continuous data were calculated based on valid cases. Inductive content analysis was undertaken to formulate categories from open-ended questions.

## Results

### Sample characteristics

There were 166 surveys completed; 114 parents and 52 patients constituting response rates of 76% and 56%, respectively. The mean age of parents was 45.1 (SD 7.59) and patients 16.4 (SD 1.85). The majority of participants were Australian born (parents: 82.5%, patients: 90.4%) and spoke English as their first language (parents: 92.0%, patients: 94.2%). Parents comprised mainly of self-described “female/woman/cis-woman” (87.5%). Patients' self-described gender identity varied; common responses included “male” (44.2%), “female”/“girl”/“woman” (34.6%), “male transgender” (5.8%) and “gender fluid”/“nonbinary”/“transgender/nonbinary” (5.8%). Families had been attending the RCHGS from 1 month to >3 years, with 58.2% attending for 12+ months. More than 80% had attended at least three appointments, and half (50.9%) were on the wait list for 6+ months.

### Description of health care received

Participants reported the health care received at RCHGS positively, with 95.6% of parents and 82.7% of patients describing this as “good” or “excellent”. Most parents and patients reported clinicians of all disciplines as helpful/very helpful (proportions ranging from 83.3% to 98.8%).

### Distress before first appointment and now

**FIG. 1.**
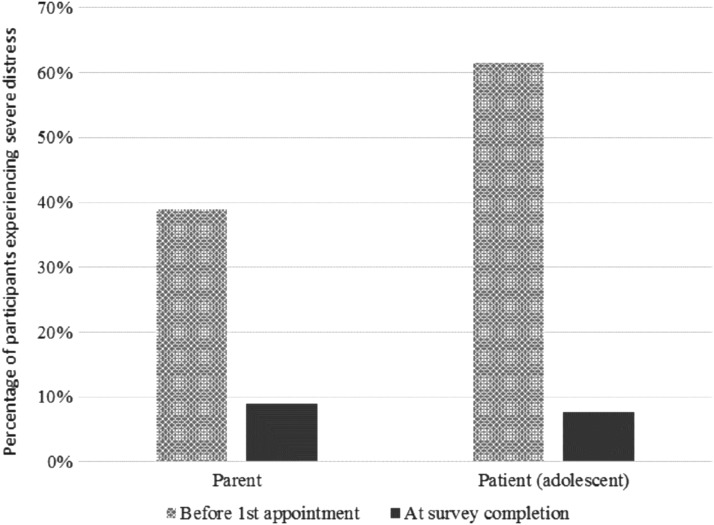
Percentage of parents and patients experiencing severe distress due to patients' gender concerns.

Figure 1 shows changes in severe distress described before first appointment and at the time of survey completion. A trend was noted whereby there was a reduced proportion of parents and patients reporting severe distress and greater proportions reporting mild distress over time.

### Satisfaction with family-centered care

The majority of participants were satisfied with the RCHGS overall (parents: 88.3%, patients: 92.3%) and would recommend the service (parents: 94.6%, patients: 88.6%). High levels of satisfaction with all aspects of family-centered care were reported (Table 1). More than 90% of patients and parents reported being satisfied/very satisfied with health staff in the areas of having knowledge in gender and maintaining confidentiality, and respect for patients' privacy by outpatient and administration services. The lowest level of satisfaction was regarding wait time for first appointment.

**Table 1.** Levels of Satisfaction with Patient and Family-Centered Practice at the Royal Children's Hospital Gender Service

	Parents							Patients						
		Dissatisfied/very dissatisfied		Neither dissatisfied nor satisfied		Satisfied/very satisfied			Dissatisfied/very dissatisfied		Neither dissatisfied nor satisfied		Satisfied/very satisfied	
	*N*	%	*n*	%	*n*	%	*n*	*N*	%	*n*	%	*n*	%	*n*
Understand young person's concerns	112	1.8	2	8.9	10	89.3	100	52	7.7	4	7.7	4	84.6	44
Understand family's concerns	112	4.5	5	9.8	11	85.7	96	52	3.8	2	15.4	8	80.8	42
Are caring and warm	112	1.8	2	8.0	9	90.2	101	52	7.7	4	7.7	4	84.6	44
Respect family's opinions and feelings	110	2.7	3	8.2	9	89.1	98	52	1.9	1	17.3	9	80.8	42
Respect young person's opinions and feelings	111	0.9	1	4.5	5	94.6	105	52	5.8	3	11.5	6	82.7	43
Have knowledge in the area of gender	110	0.0	0	6.4	7	93.6	103	52	3.9	2	5.8	3	90.4	47
Maintain young person's confidentiality	111	0.9	1	2.7	3	96.4	107	52	1.9	1	1.9	1	96.2	50
Encourage me to ask questions	110	4.5	5	8.2	9	87.3	96	52	5.8	3	9.6	5	84.6	44
Information on gender or gender management options (if provided)	85	4.7	4	9.4	8	85.9	73	49	4.1	2	14.3	7	81.6	40
Information of community gender supports and information (if provided)	86	5.8	5	19.8	17	74.4	64	42	4.8	2	28.6	12	66.7	28
Administration staff	110	7.3	8	11.8	13	80.9	89	50	8.0	4	24.0	12	68.0	34
Staff at outpatients' desk	111	4.5	5	9.0	10	86.5	96	52	1.9	1	19.2	10	78.8	41
Respect for young person's privacy by administration/outpatients	111	0.9	1	8.1	9	91.0	101	51	2.0	1	7.8	4	90.2	46
Overall satisfaction with RCHGS	111	1.8	2	9.9	11	88.3	98	52	7.7	4	0.0	0	92.3	48
Wait time for first appointment	110	33.6	37	12.7	14	53.6	59	51	29.4	15	27.5	14	43.1	22

RCHGS, Royal Children's Hospital Gender Service.

### Helpful aspects and areas for improvement (qualitative)

When asked what had been most helpful, beneficial, or valuable about RCHGS, participants expressed their appreciation of clinician's respectful and caring communication, clinicians sharing information, and the therapeutic clinician–patient relationship. Parents articulated that the process was validating and normalizing. Patients were grateful to access medical treatment. Suggestions for service improvements included additional resources to reduce wait time and increase frequency of appointments. Participants expressed requiring more detailed information during assessment and treatment. Parents stressed the need for additional support for families. Suggestions for services included family support, regional services to improve and broaden access, and assistance transitioning from pediatric to adult services. Examples of quotes reflecting these themes are provided in Table 2.

**Table 2.** Helpful Aspects of Royal Children's Hospital Gender Service and Suggestions for Improvement

Theme	Example quotes
Helpful, beneficial, or valuable aspects of the gender service:	
Appreciation of clinician's respectful communication, clinicians sharing information, and the therapeutic clinician–patient relationship	“I had so many questions about things. They were a wealth of information and provided a place where I could talk freely and openly without feeling judged. This was invaluable.” Parent
	“I find having someone so friendly and respectful to talk to really puts my mind at ease. Unlike many other appointments I have elsewhere, I look forward to coming to the appointments I make at the RCH.” Adolescent
Validating process and normalizing experience	“Someone to talk to you as though this is normal. [Clinicians] both normalised the situation, explaining it wasn't weird or strange, just another health condition that needed care.” Parent
Grateful to access medical treatment	“To gain access to professional assessment and guidance as well as reassurance that our attitudes and treatment of our child is valid and appropriate. This also gave us credibility when explaining our child to family and friends, community members and schools.” Parent
	“It's the literal only way someone my age can get hormone therapy” Adolescent
Suggestions for change/improvement:	
Additional resources	“Extra doctors so that it doesn't take so long to get an appointment.” Parent
	“The waiting time. Yes, it may not seem like very long but when your body is mutating everyday is like torture.” Adolescent
Require more detailed information during assessment and treatment	“Clearer intake process so we understand what to expect and how we can make the most of our attendance.…We have felt lost in a big system.” Parent
Additional support for families	“Separate mental health supports for parents.” Parent
Additional services that the gender service should provide;	
Family support	“Peer support for both the child and the parents and siblings” Parent
Regional services	“I think it would be good to have outreach clinics in the bigger country towns.” Parent
Assistance transitioning from pediatric to adult services	“Either see over 18s at the RCH or help with transition into adult services. I felt that the services and support dropped off when I turned 19.” Adolescent

RCH, Royal Children's Hospital.

## Discussion

This study extends our understanding of health service users' experiences of care at a multidisciplinary pediatric gender service, from parent and patient perspectives. The study revealed that patients and parents reported reduced distress having attended the RCHGS, found clinicians of all disciplines helpful, and had high levels of satisfaction with family-centered care, indicating that a multidisciplinary treatment model is accepted by this pediatric population and their families. Areas for improvement were the need to address the wait list and frequency of appointments, family support resources, and additional information provision. These findings can guide service improvements and inform other health services for this growing patient population.

In this study, most participants reported positively on the care received at RCHGS. The importance of this is twofold: first, satisfaction with health services is associated with improved health and well-being^14,15^; the synchronicity of this being imperative given the mental health vulnerabilities of this patient group, and second, it is considered an integral indicator of quality care.^12^ The level of satisfaction with RCHGS is comparable with that in adult gender services around the world.^16,18,19^

This study found that patient distress declined after attending the service, similar to studies in adult populations.^16,18^ This is an important finding in light of the service's aim to improve the mental health and well-being of children and adolescents experiencing gender dysphoria. Parent distress also improved, suggesting that the RCHGS may have a broader impact on parental well-being. This shows promise as families raising gender diverse children can experience grief,^20^ and parental support is associated with reduced adolescent distress.^21^

The need to address the waiting list was highlighted in this study; at the time there was a wait period of >12 months before patients were typically first seen by a pediatrician and mental health clinician as part of a multidisciplinary assessment clinic. Internationally, the availability of sufficiently trained specialists working in this area is limited,^4^ and when coupled with increasing referrals this results in lengthy wait times, which is a highlighted reason for dissatisfaction.^22^ Since this study, the RCHGS has introduced a single-session clinic as its standard entry point, led by the RCHGS clinical nurse consultant, which has reduced wait time for face-to-face clinical access to the RCHGS.^23^ Key components of the clinic include a biopsychosocial assessment (which is used to identify triage pathways) and provision of individualized support, education, and linkages to community services. Other gender services may benefit from implementing similar innovations. Building capacity in the health care sector through medical education and training in transgender health is imperative for ensuring optimal, accessible, and equitable care.^24,25^

There are some limitations of this study. This study used a convenience sample; satisfied individuals may be more likely to participate presenting a potential nonresponse bias.^13^ Whether this sample is representative of the RCHGS patient group and gender diverse youth in Australia is also unknown. Furthermore, this study relied on retrospective reporting, which raises potential recall bias; further prospective longitudinal research is needed. Finally, this study surveyed patients aged 12 years or older; thus, the results may not represent the entire patient population. Future efforts could focus on adapting the survey to also capture younger patients' experiences.

## Conclusion

This study demonstrated high levels of satisfaction with a pediatric gender service, confirming that a multidisciplinary assessment and family-centered care model is considered acceptable for this patient population. Areas for improvement could be targeted as this service evolves. This study's findings can be used by those developing similar services that face issues around the complexities of care for this group and ongoing demand for services.

## Abbreviations Used

RCH: Royal Children's Hospital
RCHGS: Royal Children's Hospital Gender Service
